# The Atacama toad (*Rhinella atacamensis*) exhibits an unusual clinal pattern of decreasing body size towards more arid environments

**DOI:** 10.1186/s40850-021-00090-w

**Published:** 2021-09-07

**Authors:** Felipe Durán, Marco A. Méndez, Claudio Correa

**Affiliations:** 1 Laboratorio de Sistemática y Conservación de Herpetozoos, Departamento de Zoología, Facultad de Ciencias Naturales Y Oceanográficas, Universidad de Concepción, Víctor Lamas 1290, Concepción, Chile; 2 Laboratorio de Genética y Evolución, Departamento de Ciencias Ecológicas, Facultad de Ciencias, Universidad de Chile, Santiago, Chile

**Keywords:** Bergmann’s cline, Ectotherm, Atacama Desert, Aridity gradient, Information-theoretic approach, Converse water availability hypothesis

## Abstract

**Background:**

The causes of geographic variation of body size in ectotherms have generally been attributed to environmental variables. Research in amphibians has favored mechanisms that involve water availability as an explanation for the geographic variation of body size. However, there are few studies at intraspecific level on amphibians that inhabit desert or semi-desert environments, where hydric restrictions are stronger. Here, we describe and inquire as to the causes of the geographic variation of body size in the semi-desert toad *Rhinella atacamensis*, a terrestrial anuran that is distributed over 750 km along a latitudinal aridity gradient from the southern extreme of the Atacama Desert to the Mediterranean region of central Chile. We measured the snout-vent length of 315 adults from 11 representative localities of the entire distribution of the species. Then, using an information-theoretic approach, we evaluate whether the data support eight ecogeographic hypotheses proposed in literature.

**Results:**

*Rhinella atacamensis* exhibits a gradual pattern of decrease in adult body size towards the north of its distribution, where the climate is more arid, which conforms to a Bergmann’s cline. The best model showed that the data support the mean annual precipitation as predictor of body size, favoring the converse water availability hypothesis.

**Conclusions:**

Most studies in amphibians show that adult size increases in arid environments, but we found a converse pattern to expected according to the hydric constraints imposed by this type of environment. The evidence in *R. atacamensis* favors the converse water availability hypothesis, whose mechanism proposes that the foraging activity determined by the precipitation gradient has produced the clinal pattern of body size variation. The variation of this trait could also be affected by the decreasing productivity that exists towards the north of the species distribution. In addition, we found evidence that both pattern and mechanism are independent of sex. Lastly, we suggest that behavioral traits, such as nocturnal habits, might also play an important role determining this differential response to aridity. Therefore, the support for the converse water availability hypothesis found in this study shows that amphibians can respond in different ways to water restrictions imposed by arid environments.

**Supplementary Information:**

The online version contains supplementary material available at 10.1186/s40850-021-00090-w.

## Background

Geographic variation holds a central role in evolution since it is related to the nature of species and the speciation process [1]. This variation may be present as discrete units or geographic gradients and related to the climate or the biogeographic history of the species [1, 2]. The tendencies presented as spatial gradients have been identified as ecogeographic rules [3, 4]. Among these, the relation between body size and environmental variables stands out [5], since body size is a trait strongly linked to the ecology and evolution of organisms [6]. The most studied generalization about geographic variation of body size is Bergmann’s rule [7–14]. This rule proposes that, among closely related endotherms, body size increases with lower temperatures since animals with larger body size have less heat exchange with the environment (lower area-volume ratio), and thus will be able to conserve heat in colder areas [7] (translation in supplementary material of [15]). However, latitude has traditionally been the predictor of body size due to its high correlation with temperature at large scales [16, 17].

Distinction between pattern and mechanism is key when dealing with ecogeographic rules [16, 18]. In the case of the Bergmann’s rule, it is necessary that temperature and its conservation mechanism be identified in the study system. When the pattern of increase in body size with latitude is present, but the mechanism is different to heat conservation, it has been proposed to use the term Bergmann’s size cline instead [19]. Thus, several mechanisms have been proposed to explain this and other geographic patterns of body size in ectotherms, among which the most frequent are related to heat balance [20, 21], water availability [22, 23], resource availability [24, 25], environment seasonality [26] and life-history attributes [27]. This variety of proposed mechanisms highlights the difficulties associated with providing conclusive evidence for any of them [28].

The study of these mechanisms in amphibians has favored explanations mainly related to water availability [22, 23, 28–30], given their strong dependence on water due to the permeability of their skin [31]. This has led to the formulation and evaluation of mechanisms that predict different body size variation patterns related to hydric constrains (Table 1). One important contribution in this context was to consider that thermal and water balance are related (e.g. water loss from the surface leads to a simultaneous heat loss). This relationship suggests that a better descriptor of body size would involve a measure of water loss through the skin, as well as the energy present in the environment (potential evapotranspiration) instead of temperature or water availability by themselves [29]. However, these hypotheses have seldom been evaluated formally at intraspecific level in species distributed in aridity gradients.

**Table 1:** Hypotheses proposed in literature to explain the geographic variation of body size in amphibians

Hypothesis	Predictor variable	Predicted relation with body size	Mechanism(s) (Key references)
Water availability	Mean annual precipitation (BIO12)	Negative	A lower area-volume relation given by greater body size will produce less surface for water loss ([22])
Converse water availability	Mean annual precipitation (BIO12)	Positive	Amphibian activity is strongly related to high water availability and humid periods, allowing more foraging time that promotes greater body size in areas with more precipitation ([51])
Water conservation	Potential evapotranspiration (PET)	Negative	Water loss leads to heat loss, thus thermal balance is intimately linked to water balance in amphibians. Thus, a lower area-volume relation given by greater body size reduces the capacity of the environment to remove water and heat from the body surface ([29])
Seasonality	Temperature seasonality (BIO4)	Negative	The individuals of populations with longer times of favorable activity are larger than those with shorter and more fluctuating times ([25])
Starvation resistance	Temperature seasonality (BIO4)	Positive	Greater body size allows greater energy reserves to withstand periods of lower resource availability compared to smaller body size ([10]), since the rate of energy storage is greater than that of consumption (see discussion in [52])
Primary productivity	Normalized Difference Vegetation Index NDVI	Positive	Greater food availability provides the possibility to reach larger body size ([21, 24])
Heat balance	Mean annual temperature (BIO1)	Negative	Greater body size allows more thermal inertia, providing advantages to thermoregulating ectotherms with larger body size in cold climates. Inversely, in thermoconforming ectotherms small body size is favored in colder zones, since they heat more quickly ([21])
Size-temperature rule	Mean annual temperature (BIO1)	Negative	The maturation times in most ectotherms are longer in cold climates, which results in greater body size ([53])

Along with the predictor variables, corresponding bioclimatic variables used in the information-theoretic approach are shown in parentheses

Other important factors that influence geographic tendencies of body size in amphibians are their geographic context and habitat preferences [29, 32–34]. For example, amphibians from desert or semi-desert environments will likely respond to the pressures imposed by aridity and large daily temperature variation with morphological and/or behavioral adaptations [35]. By contrast, in lineages of aquatic amphibians it is expected that the effect of water availability on body size will be irrelevant [29]. Thus, amphibians of desert and semi-desert environments offer the opportunity to study the effects of water availability on the geographic variation of body size. Nevertheless, there are still few intraspecific studies that have used species that inhabit this kind of environment as models [22, 36, 37] and none have explicitly evaluated ecogeographic hypotheses.

**Fig. 1:**
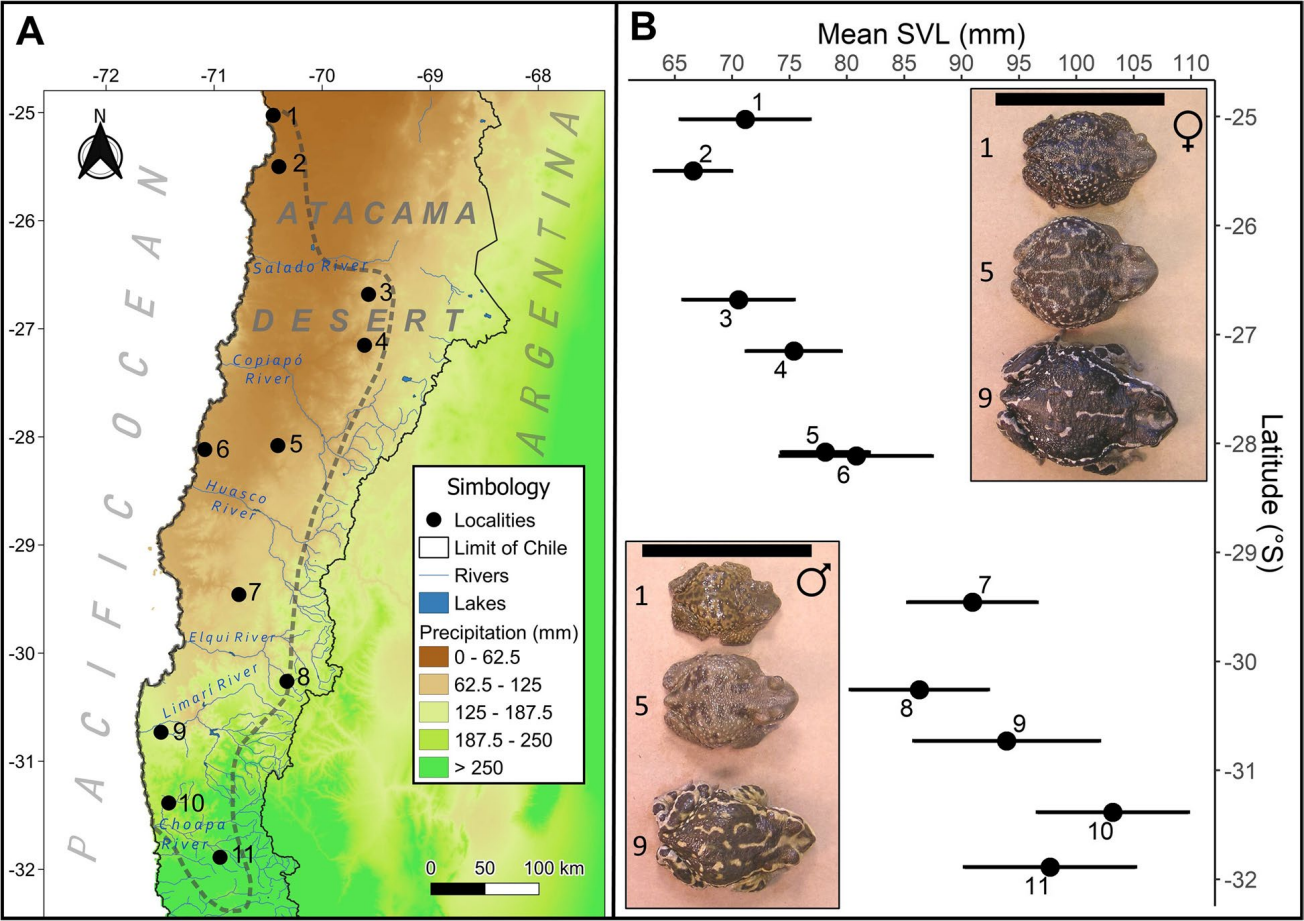
Sampling localities and body sizes (snout-vent length, SVL) of *Rhinella atacamensis*. **A** Geographic location of the 11 localities included in this study (numbers given in Table 2). Colors represent different levels of precipitation (mean annual precipitation) and the dashed line delimits the approximate distribution of the species. **B** Geographic variation of body size (mean and standard deviation of SVL of each locality) as a function of latitude (R^2^ = 0.91). The photographs show the differences in size of males and females of three representative localities (numbered according to Table 2). Black bars indicate 10 cm. The map is own elaboration

The Atacama Desert, located in the extreme north of Chile, is considered one of the driest places on the planet [38]. Although it is postulated that the extreme aridity of this desert originated in the late Miocene [39], its current climatic conditions would have been installed during the Plio-Pleistocene [40]. The only amphibian that has colonized the extreme south of the Atacama Desert is the Atacama toad (*Rhinella atacamensis*), a terrestrial species endemic to the semi-desert zone of Chile. Its distribution extends latitudinally over more than 750 km from the desert (25°S) to the Mediterranean zone of the center of the country (32°S), in a climatic transition zone in which precipitation (means of the localities of the extreme north and south vary between 14 to 228 mm/year; Fig. 1A) and productivity gradually increase southwards, while seasonality is accentuated [41, 42]. In much of its distribution is sympatric with *Pleurodema thaul*, but only *R. atacamensis* is distributed in the most arid part of this range (25–27°S), hence it is considered a true desert inhabitant [43–45]. It was originally thought that this species was restricted to a few isolated localities between Paposo (Antofagasta Region) and the Huasco River (Atacama Region) (25–29°S), where it lives closely associated with water systems (some of which have a very small extension), but subsequently its distribution was considerably expanded to the south (~ 32°S, Coquimbo Region; reviewed in [45]).

*Rhinella atacamensis* has a notable geographic variation in body size and color pattern [44–48]. Almost 60 years ago, the first studies of the populations in the extreme north of its range revealed differences among them in both coloration and sexual dimorphism (less evident in the Paposo population), and in body size [43, 44]. The degree of intraspecific variation in coloration and body size is even greater when populations south of the distribution are considered (Coquimbo Region, ~ 30–32°S [47, 51]). The adults of the southern populations are larger and have different dorsal coloration ([46], represented by individuals of locality 9 in Fig. 1B) than those of the extreme north, different enough for Cei [43, 44] to consider them a different species (*R. arunco*) in his seminal studies of the genus *Bufo* in Chile (now *Rhinella*). Nowadays, the taxonomic status of the species is clear [50] and its geographic distribution with respect to its sister species (*R. arunco*) is better known [45], but the amount and form of the variation of body size (e.g. clinal pattern or discrete groups) has not been studied in its entire distribution.

The high level of phenotypic variation among the populations of *R. atacamensis*, which are distributed along an extensive aridity gradient, offers an interesting opportunity to study the causes of intraspecific body size variation in amphibians. Firstly, geographic variation of body size in this species is described through its entire distribution range. Then, using data from representative localities and an information-theoretic approach, we evaluate the predictions derived from the hypotheses related to water availability as the principal mechanism. However, since body size is a complex trait and its geographic variation may be influenced by multiple factors [1], hypotheses involving temperature, resource availability and seasonality (Table 1) are also considered. We hypothesize that precipitation will be the main predictor of body size in the Atacama toad, considering that this factor becomes limiting towards the north of its current distribution (Fig. 1A) and the antiquity of the aridity gradient where this species is distributed.

## Results

The overall mean of the snout-vent length (SVL) of the 315 adult individuals (190 males, 125 females) was 84 mm (S.D. = 13.2 mm); mean SVL was 83.2 mm (± 13.9 mm) in males and 85.1 mm (± 12 mm) in females, which were not significantly different (W = 10.382; *p* = 0.0591). Student’s t-tests showed that there are differences between male and female SVL in five locations, where females were larger (Table 1 in Additional file 2). The smallest mean SVL for males and females was found in Las Breas (65.4 and 67.8 mm, respectively) and the largest mean SVL for males and females was in Canela Alta (102.5 mm and 109.1 mm, respectively; Table 1 in Additional file 2). It should be noted that there was no overlap in the ranges measured in these two localities, and that there is only a small overlap in the ranges of the extreme localities (Paposo and Palquial) (Table 2). In all localities (except Mostazal) the smallest sizes correspond to males, while the largest individuals were always females (except Paposo; Table 1 in Additional file 2).

**Table 2:** Sample sizes, body size (mean snout-vent length, SVL ± standard deviation, S.D.), range of SVL, mean annual precipitation (Pp) and coordinates of the sampled localities of *Rhinella atacamensis*, ordered from north to south

N°	Locality	Individuals by locality	SVL ± S.D. (mm)	Range (mm)	Pp (mm)	Latitude (°S)	Longitude (°W)
1	Paposo	48	71.1 ± 5.6	55.2 – 85.3	14	-25.026	-70.453
2	Las Breas	10	66.6 ± 3.3	61.1 – 73.0	17	-25.500	-70.401
3	Mostazal	27	70.6 ± 4.8	56.4 – 77.6	47	-26.682	-69.571
4	Vega Cebollar	42	75.4 ± 4.1	68.1 – 84.0	34	-27.153	-69.608
5	Quebrada Los Sapos	23	78.1 ± 3.8	71.9 – 88.0	40	-28.080	-70.410
6	Llanos de Challe	21	80.8 ± 6.6	73.0 – 97.7	40	-28.117	-71.086
7	Los Pajaritos	34	91.0 ± 5.6	82.3 – 100.0	64	-29.458	-70.771
8	Cochiguaz Alto	9	86.3 ± 6	80.5 – 98.5	104	-30.261	-70.326
9	Socos	25	93.9 ± 8.1	78.0 – 107.8	127	-30.731	-71.491
10	Canela Alta	30	103.1 ± 6.6	88.3 – 112.8	167	-31.386	-71.420
11	Palquial	46	97.7 ± 7.4	82.4 – 110.8	228	-31.888	-70.945

**Fig. 2:**
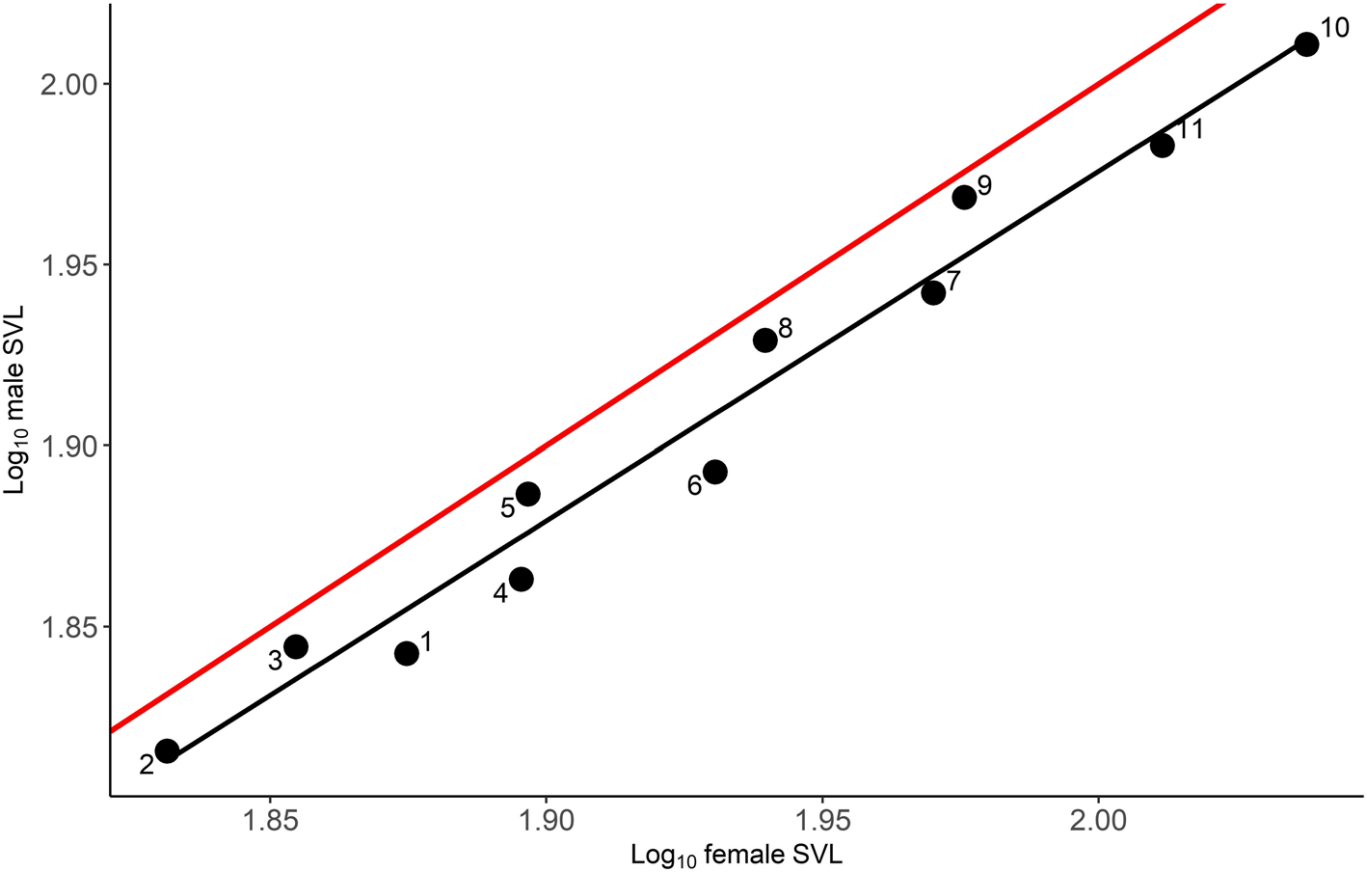
Model II regression of mean body size (snout-vent length, SVL) of males on females of *Rhinella atacamensis*. Circles represent the combined means of males and females per locality. The red line indicates isometry (slope equal to 1). The black line is the model II regression slope (slope = 0.979, *p* = 0.725). Localities are numbered according to Table 2

Model II regression did not show a significative difference from the isometric slope (Fig. 2; slope = 0.979, *p* = 0.725), indicating that the degree of sexual dimorphism does not change with body size. Therefore, males and females show a similar pattern of decrease of body size with latitude (Table 1 in Additional file 2). Considering this, and that the aim was to assess the pattern of variation in body size at the species level, regardless of sex, Fig. 1B shows the relationship between mean body size of total data (both sexes) by locality and latitude.

**Fig. 3:**
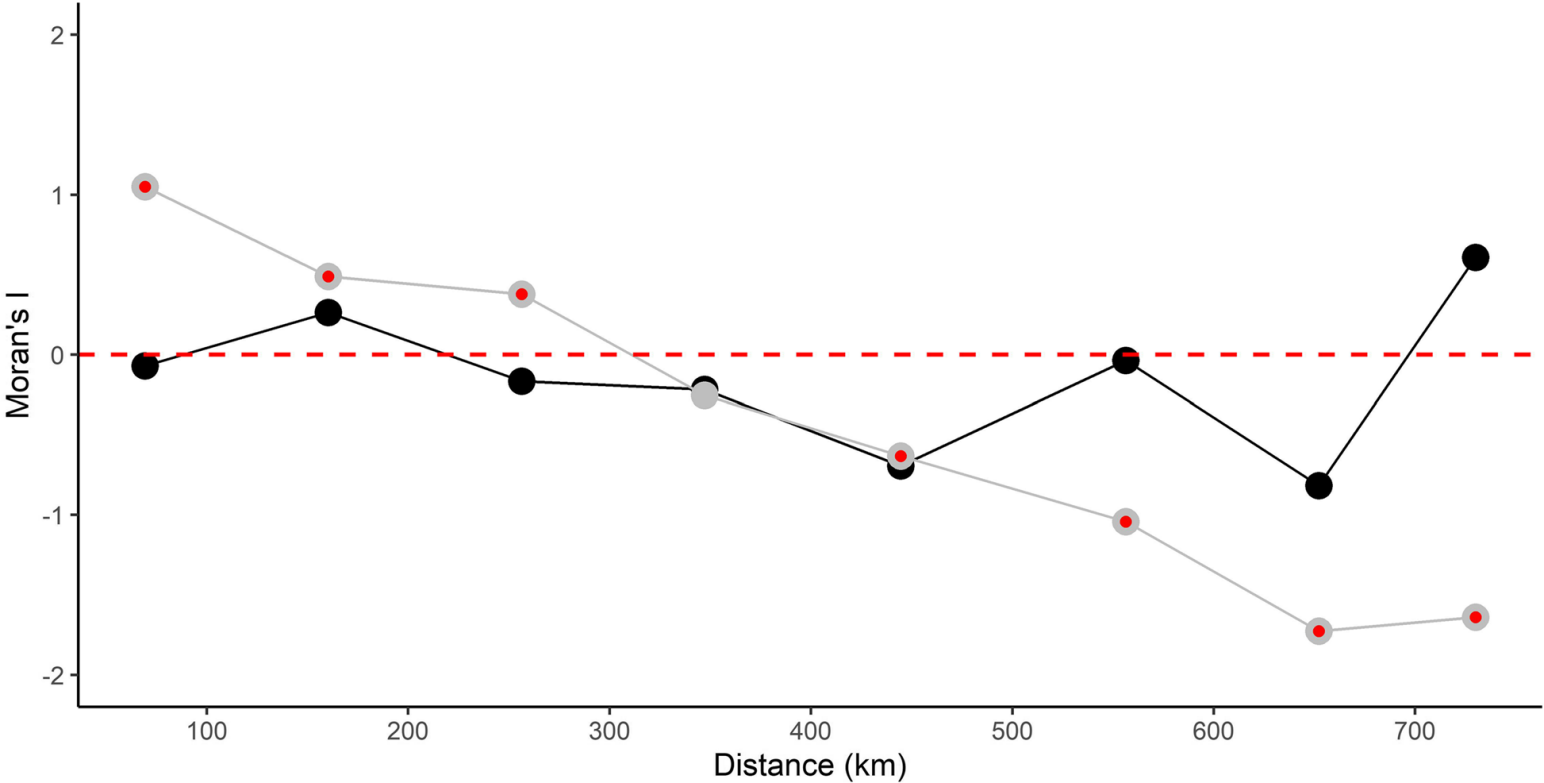
Spatial correlograms of Moran’s I of adult mean SVL (grey circles and lines) and the residuals of the best model (black circles and lines) for eights distance classes (in kilometers). Red filled circles indicate statistical significance (*p* < 0.05)

A clear pattern of decrease in SVL was observed northwards of the species distribution (R^2^ = 0.91, slope = 4.92), which is consistent with a Bergmann’s body size cline (Fig. 1B). According to Moran’s I, body size was positively autocorrelated at short distances and negatively at long distances grouping sexes by locality (Fig. 3) and considering each sex separately (Figs. 1 and 2 in Additional file 2), which indicates that only nearby populations had similar body size. This is concordant with the pattern of clinal geographic variation observed in Fig. 1B.

The best model ranked with AICc for all data by locality (Table 3) and the best one considering each sex separately (Table 1 in Additional file 2) included only mean annual precipitation (BIO12) as predictor. In each of these analyses, the mean annual precipitation alone explains over 73% of the body size variation (Table 3 and Table 2 in Additional file 2). The residuals of these models were not spatially autocorrelated (Monte Carlo permutation test, *p* < 0.05), indicating that there are no biases in our results.

**Table 3:** Linear regression models of climate variables on body size of adult males and females of *Rhinella atacamensis* (mean snout-vent length by locality), ranked by the values of AICc, from the best to the worst model

	Model	Adjusted R^2^	K	AICc	∆AICc	AICw
1	BIO12 ( +)	0.73	3	78.80	0.00	0.39
2	BIO12 ( +), PET (-)	0.80	4	79.36	0.56	0.30
3	NDVI ( +)	0.65	3	81.91	3.11	0.08
4	B12 ( +), NDVI (-)	0.80	4	82.44	3.64	0.06
5	NDVI ( +), B1 (-)	0.71	4	83.49	4.69	0.04
6	B12 ( +), BIO4 ( +)	0.71	4	83.72	4.92	0.03
7	B12 ( +), B1 (-)	0.70	4	84.02	5.22	0.03
8	NDVI ( +), B4 (-)	0.66	4	85.54	6.74	0.01

Only models with low differences in AICc values relative to the best model (Δ*i* < 7) are shown. For each model, the predictor variable name (with its respective regression coefficient sign), R^2^ adjusted, estimated number of parameters (K), AICc values, delta AICc (Δ*i*) and Akaike weights (AICw) are shown. The environmental variables were mean annual temperature (BIO1), temperature seasonality (BIO4), mean annual precipitation (BIO12), normalized index of vegetation difference (NDVI) and potential evapotranspiration (PET)

**Fig. 4:**
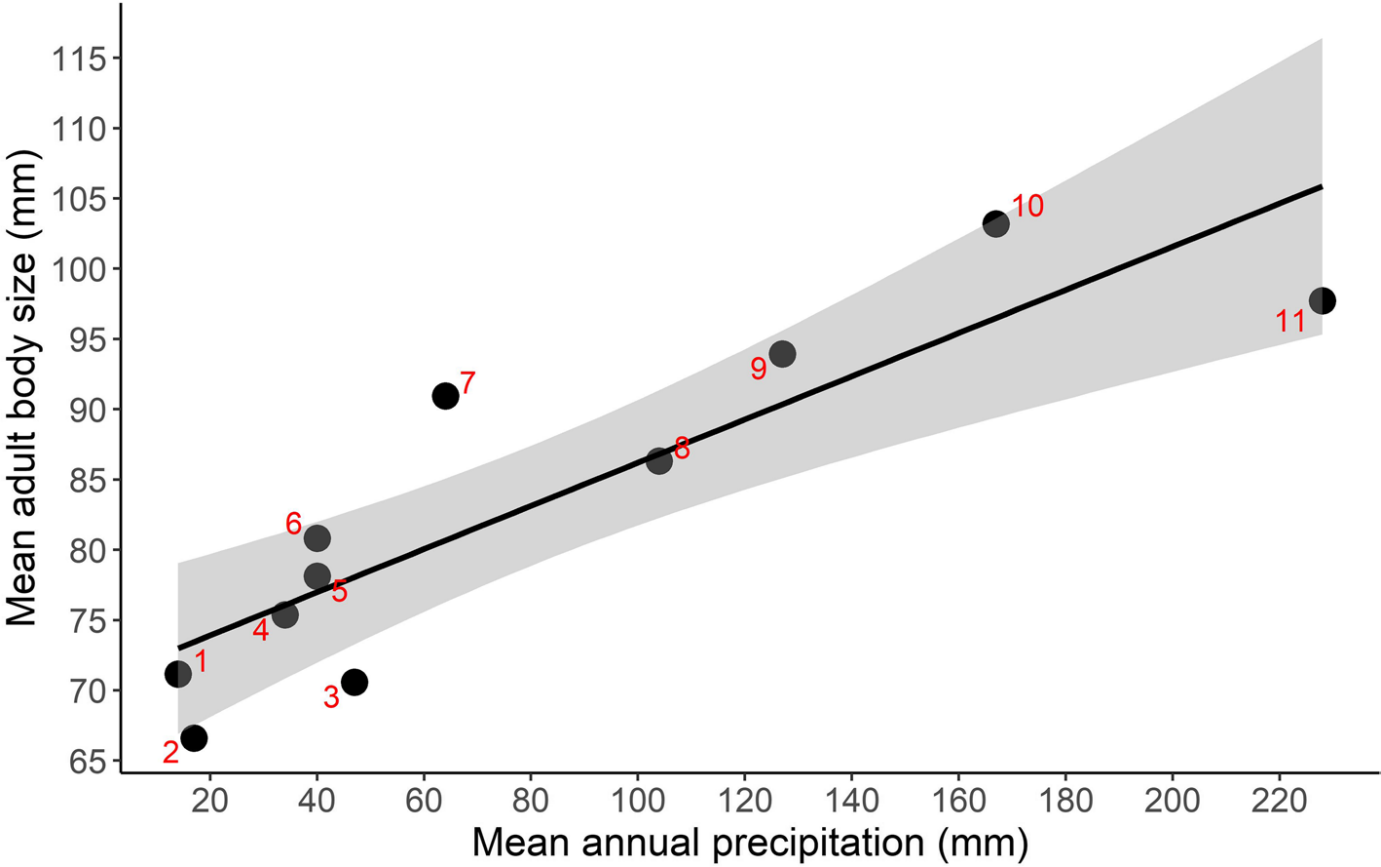
Linear regression of mean annual precipitation on mean adult body size of the 11 localities of *Rhinella atacamensis* studied. The grey area indicates the 95% prediction interval of the mean. Localities are numbered according to Table 2 with red numbers

In addition, the second-best models of the pooled and sex-separated data by locality included the variable BIO12 together with the potential evapotranspiration (PET) (Table 3 and Table 2 in Additional file 2). Therefore, the main bioclimatic variable to explain the variation in body size in *R. atacamensis*, regardless of sex, is precipitation (Fig. 1 in Additional file 2). In fact, the linear regression of Fig. 4 shows that precipitation explains an important part of body size variation in the species (R^2^ = 0.76, *p* < 0.01).

**Fig. 5:**
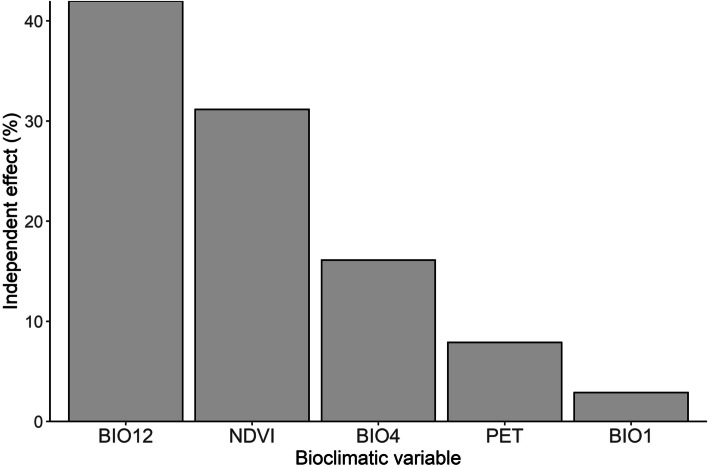
Percentage of independent effect of the five environmental variables included in the 32 models evaluated, calculated using hierarchical partition. The environmental variables are: mean annual temperature (BIO1), temperature seasonality (BIO4), mean annual precipitation (BIO12), Normalized Difference Vegetation Index (NDVI) and potential evapotranspiration (PET)

In general, very similar results were obtained with the data grouped or separated by sex, so only those obtained with all the data are described below. The first and second models explained a substantial amount of evidence (0.39 and 0.30, respectively), which indicates that both models provide similar support to the data [55]. According to evidence ratios, the best model was 1.32 and 4.73 times more probable than the second and third highest ranked models. Considering the independent contribution of each environmental variable, mean annual precipitation explained 42% of the body size variability of Atacama toad, followed by NDVI with 31.2% (Fig. 5). All results of analyses for each sex are shown in the Additional file 2.

## Discussion

Previous inter and intraspecific studies on geographic variation in amphibians have shown that they respond to water restrictions imposed by driest habitats through an increasing of body size [22, 28, 29, 34, 36, 37, 55, 56]. In contrast, our results show a pattern of decreasing of body size in *R. atacamensis* northward of its distribution, as the environment becomes more arid (Fig. 1A). Moreover, this pattern fits a Bergmann’s size cline (Fig. 1B). Therefore, we corroborate the observations of Cei [43, 44] in the northern part of the species distribution, where a clinal increase with latitude was described, and we show that this pattern extends throughout its entire distribution and is directly associated with precipitation (Fig. 4 and Fig. 1 in Additional file 2). The presence of this variable in the best models (Table 3; see results separated by sex in Table 1 in Additional file 2) and its high independent effect (Fig. 5) favor the converse water availability hypothesis (Table 1). Moreover, the results show that there is an isometric pattern of sexual dimorphism (no evidence of Rensch’s rule [54], Fig. 2) and that both males and females have responded identically to the decreasing of precipitation northwards of the species distribution (Fig. 1 in Additional file 2).

Considering the biogeographic scenario of *R. atacamensis*, the activity of this species towards the north of its distribution would be lower due to the increase in aridity, impacting negatively in its foraging time, resulting in smaller body sizes. Experimental evidence in ectothermic vertebrates points in the same direction. Foraging activity is limited in conditions of lower environmental humidity [57], as well as less efficient [58], which in turn affects net energy gain with a subsequent low growth rate [59]. In fact, the reduction in the foraging efficiency and activity would explain the dwarfism in two species of terrestrial toads that inhabit sandy substrates [60]. Furthermore, models predict that when growth rate is reduced by a decrease in food quality, body size also decreases [61]. For example, limited activity and foraging opportunities in *Pelobates cultripes* resulted in a lower grow rate and smaller body sizes [62]. Similar patterns have been observed in a snake species [63] that inhabits arid regions, but in this case the explanation have been focused on food availability more than foraging abilities. Both explanations are related and, although they possess different underlying mechanisms (Table 1), both may be affecting the Atacama toad in a non-exclusive way. In this species, the NDVI is positively related to body size, it has the second largest independent effect (Fig. 5) and it is included in the third best model in the AICc ranking (Table 3). Considering this variable, the decrease in foraging activity in the Atacama toad could be affected at the same time by a reduction in foraging area and/or food supply.

The preponderance of precipitation as the main predictor of body size in *R. atacamensis* is consistent with previous intraspecific studies of amphibians that inhabit arid regions and precipitation gradients [22, 34, 36, 37, 55, 56]. However, a converse pattern of body size variation, like that exhibited by the Atacama toad, rarely has been described in amphibians. Interestingly, one of the few examples comes from a co-distributed species (from 27°S to the south), *Pleurodema thaul* [64]. In that study, the pattern was explained arguing that higher minimum temperatures and lower precipitation northwards of its distribution would have reduced hydroperiods, resulting in small postmetamorphic sizes [64]. This mechanism seems less plausible for *R. atacamensis* because temperature was not an important bioclimatic variable to explain its variation of body size (BIO1 in Table 3 and Fig. 5), but could be a non-exclusive explanation. However, the parallel pattern in these co-distributed species provides an important opportunity to investigate in a common garden design the ultimate causes of body size variation of both species [65]. In addition, the parallel patterns in sympatric populations of different species suggest that these clines may be adaptive [66], suggesting that similar processes could be producing them.

The converse pattern described in the present study suggests that the response does not directly involve the water economy (i.e. water availability and conservation) as expected under an aridity gradient. Thus, other ecological processes could be affecting the body size variation or could even be more important than the effect of water conservation [67]. Although very little is known about the natural history of *R. atacamensis*, there are some aspects of habitat and behavior that could be important in this context. Populations in the northern distribution of the species (north of 29°30’S) inhabit mainly isolated streams with permanent flow [45] and have associated behaviors such as hiding under rocks in running water or near the edges [44]. The species also has nocturnal habits [68], which allows it to avoid the greater dehydration rates produced by diurnal temperatures [35]. The lack of evidence in favor of hypotheses related to temperature and water economy suggests that this type of explanation is important in the case of *R. atacamensis* (Table 3).

Data collected in the present study allowed to evaluate sex differences through the entire distribution the *R. atacamensis*. Although it was not the principal aim of this study, we were able to reevaluate some conclusions from the seminal studies of Cei [43, 44] and to compare them with new studies [68]. For instance, the sexual dimorphism skewed towards females found in Llanos de Challe (28°S [68]) was confirmed. However, the pattern of sexual dimorphism is isometric when comparing populations of all its distribution (Fig. 2). The differences in sexual dimorphism between localities could be reflecting different processes occurring at microhabitat level [69, 70] or could be due to the low number of samples in some localities (see Table 1 in Additional file 2). To evaluate this possibility at different spatial scales, we recommend using a substantially larger number of samples of both sexes and carrying out field studies such as that of Pincheira-Donoso et al. [68] in other localities. We highlight that even with differences between some localities (Table 1 in Additional file 2), the pattern of variation in body size through the precipitation gradient was found to be similar in both sexes (Fig. 1 in Additional file 2) and that the same ecogeographic hypothesis explained the pattern regardless of sex (Table 1 in Additional file 2).

Although correlations and explicit evaluation of multiple hypotheses are useful to identify the environmental factors that may be modulating the variation of traits such as body size, experimental studies are required to determine the underlying mechanisms and directly evaluate the genetic component of geographic variation [65]. However, the historical persistence of the aridity gradient, directly linked to the antiquity of the Atacama Desert, the parallel clinal pattern exhibited by *P. thaul* [64] and the ancestral distribution of *R. atacamensis* inferred by the distribution of its sister species *R. arunco*, which replaces it to the south (~ 32–38°S [46, 47]), suggest that the body size cline of *R. atacamensis* would have been an adaptive response to more arid conditions as its populations expanded further north. In fact, the current distribution ranges of both sister species allow giving a spatial direction to the process of body size decreasing in *R. atacamensis* but the time frame in which this process occurred is unknown.

## Conclusion

We described an intraspecific clinal pattern of geographic variation in body size contrary to that expected according to the literature of amphibians that are distributed in desert or semidesert environments. This is the clearest example of this type of cline (i.e., Bergmann’s size cline) described so far in amphibians, as well as the only case where the converse water availability hypothesis is favored; it should be noted that these results are independent of sex.

Moreover, this is the first study in amphibians that inhabit desert and/or semidesert environments where the putative mechanisms (i.e., ecogeographic hypotheses) were explicitly evaluated in an approach of multiple competing hypotheses. Hence, the converse water availability hypothesis emerges as an alternative to the water availability hypothesis, showing that amphibians can respond in different ways to cope with water restrictions imposed by arid environments.

## Methods

### Sampling

The SVL of 315 adults of *R. atacamensis* from 11 representative localities of its entire distribution were measured (Fig. 1A). Most measurements were made in situ by the same person (individuals were measured, photographed, and released at the capture sites), but specimens from the DBGUCH (Universidad de Chile) and MZUC (Universidad de Concepción) collections were also included. Measurements were made with a digital caliper with 0.01 mm precision and then were rounded to one decimal place. The field campaigns were performed during the reproductive season, which takes place over a few weeks between August and November, depending on the location (C. Correa and M. Méndez, personal observations). The searches for the individuals generally began a few minutes before the sunset (approximately at 19:30 h), lasting until midnight. The southern limit of the distribution of *R. atacamensis* is not clear, since around 32°S there is a zone of hybridization with its sister species *R. arunco* [49], thus sampling was extended only to the Choapa River watershed (Palquial) to include only pure populations of *R. atacamensis*. Individuals were sampled in each locality in the same stream system within a maximum distance of 4 km (Palquial), except for Llanos de Challe, in which we included a few individuals from another site 22.5 km east (Canto del Agua) located in the same watershed. The sampled localities are shown on the map in Fig. 1 within a dashed line that represents the approximate distribution range of the species. This map was prepared by the authors using the QGIS program [71]. The sex and maturity of the individuals were determined using external characters and the presence (males) or absence of vocal activity. Adult males have nuptial pads on fingers one and two of the forelimbs, and generally have yellowish background color and smooth skin. Adult females generally have a whitish color with marked dark patches, skin with small spines in the dorsal area and more robust contexture [43, 44]. Data used in this study are show in Additional file 1.

### Statistical analyses

Data normality was tested by sex, locality, and for each sex by locality with Shapiro–Wilk tests. Then, because the data of males and females were not normally distributed (Shapiro–Wilk of males: W = 0.941, *p* < 0.05; females: W = 0.964; *p* < 0.05), we examined sexual dimorphism in all samples using Mann–Whitney U tests. Differences in SVL of males and females within each locality were evaluated with Student’s t-tests. In addition, to evaluate how the degree of sexual dimorphism varies with body size, a major axis regression (model II) was performed fitting the log_10_ of mean body size of males and females [72]. This was compared to a slope equal to one, which represents the null hypothesis of isometry. The model II regression was performed in the smatr package [73]. This analysis also allowed us to evaluate another ecogeographical rule, Rensch’s rule [54].

Mean, minimum and maximum SVL for each locality were found to be highly correlated in all comparisons between pairs of variables (Pearson’s r > 0.89, *p* < 0.001), thus it was decided to use only the mean SVL in analyses. This allows comparison with studies of other anuran species, since mean SVL has often been used in studies of intraspecific geographic variation of body size (e.g. [52, 74]). Only the data from locality of Los Pajaritos was not normally distributed (W = 0.932, *p* = 0.04). Then, a linear model using mean SVL by locality and latitude as variables was obtained to evaluate the form and magnitude of geographic variation of body size in *R. atacamensis*.

### Environmental variables and hypotheses testing

Geographic coordinates of each locality were used to obtain the climatic variables from the climate surfaces constructed by [75]. These surfaces were constructed with monthly temperatures and precipitation from 1950 to 2000; they are available with a spatial resolution of 1 km^2^. The relation between the environmental variables and body size was analyzed to determine which variable better explains the geographic variation of body size, as shown in Table 1. The Normalized Difference Vegetation Index (NDVI) provides values which are highly correlated with photosynthetic mass and primary productivity [76]. The NDVI data, available with a spatial resolution of 30 arcseconds, were downloaded from [77]. Then, the maximum NDVI for each locality was obtained. Potential evapotranspiration (PET) was obtained from the CGIAR-CSI Soil–Water Balance Database [78] according to the proposal of [29]. The package Raster 3.4.5 [79] was used to extract the values of the climate variables.

We used an information-theoretic approach [80] to identify the potential mechanisms that have produced the geographic variation of body size in *R. atacamensis*. For this, bioclimatic data, NDVI and PET were used as predictor variables, generating 32 candidate models of simple linear regression (multiple with more than one predictor) for each of the six hypotheses (Table 3), considering all possible combinations of bioclimatic variables (excluding interactions). The models were evaluated using the Akaike Information Criterion corrected for small sample sizes (AICc [81]) and comparing the AICc value of each model with the minimum AICc (∆AICc) [80]. Rule of thumb was applied as suggested by [81] to report the best models, which indicates that models have considerably less support (∆AICc < 7) or substantial support (∆AICc < 2). We also used the Akaike weights (AICw) to evaluate the uncertainty of each model [80]. Evidence ratios were included to compare the relative likelihood of the models (*w*_*a*_/*w*_*b*_; where *w*_*a *_is the likelihood of model a and *w*_*b *_is that of model b [82]). Considering that male and female may respond differently to climatic variables (e.g. [23]), the AIC analyses were performed also by sex.

The relative contribution of the environmental factors on body size was assessed with an analysis of hierarchical partitioning [83, 84] considering mean of body size of all data (males and females) by locality. This analysis allows independent identification of the percentage of variation explained by each causal factor [83, 85, 86], eliminating the problems produced by multi-collinearity. For this we used the package hier.part 1.0.4 [87].

Spatial autocorrelation of body size and residuals of the best model were assessed using Moran’s I with a Monte Carlo permutation test with 199 permutations for significance evaluation, which was done using the package ncf 1.2.9 [88]. Then, spatial correlograms were created for eight distance classes for each sex and total data by locality. All analyses were performed in the R program (version 4.0.3) [89].

## Supplementary Information

**Additional file 1.** Raw data used in this study.

**Additional file 2**. Results of the analyses by sex.

## Data Availability

Dataset used in this study are available in Additional file 1.

## Abbreviations

SVL: Snout-vent length
BIO1: Bioclimatic variable 1
BIO4: Bioclimatic variable 4
BIO12: Bioclimatic variable 12
PET: Potential evapotranspiration
NDVI: Normalized Difference Vegetation Index
S.D.: Standard Deviation
Pp: Mean annual precipitation
AICc: Akaike Information Criterion corrected for small sample size
K: Number of parameters
AICw: Akaike weights
MZUC: Museo de Zoología de la Universidad de Concepción
DBGUCH: Herpetological Collection of the Departamento de Biología Celular y Genética of the Universidad de Chile
