# Supplementary material for: The Atacama toad (Rhinella atacamensis) exhibits an unusual clinal pattern of decreasing body size towards more arid environments
Source: BMC Zool. 2021 Sep 7;6:25. doi: 10.1186/s40850-021-00090-w (PMC10127348; doi:10.1186/s40850-021-00090-w)
Supplement: Supplementary file 1 — Additional file 1. Raw data used in this study. [file 40850_2021_90_MOESM1_ESM.docx]

Additional file 2

**Table 1.** Mean and ranges of body size (SVL) of males and females of *Rhinella atacamensis* from sampled localities across its distribution range (ordered from north to south). The geographic coordinates are shown in Table 2 of the main text.

| **Female** | | | | | **Male** | | | |
| --- | --- | --- | --- | --- | --- | --- | --- | --- |
| Locality | N | Mean  (mm)  ± S.D. | Min  (mm) | Max  (mm) | N | Mean  (mm)  ± S.D. | Min  (mm) | Max  (mm) |
| 1. Paposo | 14 | 74.9 ± 4.46 | 65.1 | 80.0 | 34 | 69.6 ± 5.36 | 55.2 | 84.3 |
| 2. Las Breas | 5 | 67.8 ± 3.80 | 63.1 | 73.0 | 5 | 65.4 ± 2.66 | 61.1 | 68.2 |
| 3. Mostazal | 11 | 71.6 ± 6.12 | 56.4 | 76.9 | 16 | 69.9 ± 3.77 | 62.2 | 77.6 |
| 4. Vega Cebollar | 18 | 78.6 ± 3.37 | 71.1 | 84.0 | 24 | 73.0 ± 2.71 | 68.1 | 79.7 |
| 5. Quebrada Los Sapos | 14 | 78.8 ± 4.21 | 71.9 | 88.0 | 9 | 77.0 ± 2.95 | 72.7 | 82.5 |
| 6. Llanos de Challe | 8 | 85.2 ± 7.75 | 73.0 | 97.7 | 13 | 78.1 ± 4.12 | 73.0 | 85.5 |
| 7. Los Pajaritos | 20 | 93.3 ± 5.74 | 83.2 | 100.0 | 14 | 87.5 ± 3.24 | 82.3 | 93.0 |
| 8. Cochiguaz Alto | 6 | 87.0 ± 6.36 | 80.9 | 98.5 | 3 | 84.9 ± 6.19 | 80.5 | 92.0 |
| 9. Socos | 15 | 94.6 ± 8.79 | 78.0 | 107.8 | 10 | 93.0 ± 7.18 | 79.6 | 103.7 |
| 10. Canela Alta | 3 | 109.1 ± 3.31 | 106.5 | 112.8 | 27 | 102.5 ± 6.55 | 88.3 | 110.9 |
| 11. Palquial | 11 | 102.7 ± 6.11 | 88.7 | 110.8 | 35 | 96.2 ± 7.19 | 82.4 | 110.6 |

**Table 2**. Linear regression models between climate variables and mean body size (snout-vent length*,* SVL) of adult males and females by locality of *Rhinella atacamensis*, ranked by the values of AICc, from the best to the worst model.

| Sex | Model | Adjusted R² | K | AICc | | ΔAICc | AICw |
| --- | --- | --- | --- | --- | --- | --- | --- |
| Male | BIO12 | 0.76 | 3 | 77.52 | | 0.00 | 0.38 |
|  | BIO12, PET | 0.83 | 4 | 77.74 | | 0.22 | 0.34 |
|  | BIO12, NDVI | 0.78 | 4 | 80.92 | | 3.40 | 0.07 |
|  | NDVI | 0.67 | 3 | 81.18 | | 3.66 | 0.06 |
|  | NDVI, B1 | 0.74 | 4 | 82.34 | 4.82 | | 0.03 |
| Female | BIO12 | 0.73 | 3 | 80.71 | 0.00 | | 0.39 |
|  | NDVI | 0.79 | 3 | 81.98 | 1.27 | | 0.21 |
|  | BIO4, BIO12 | 0.67 | 4 | 82.65 | 1.94 | | 0.15 |
|  | BIO4 | 0.75 | 3 | 83.71 | 3.00 | | 0.09 |
|  | BIO12, NDVI | 0.71 | 4 | 85.43 | 4.73 | | 0.04 |
|  | BIO12, PET | 0.70 | 4 | 85.65 | 4.94 | | 0.03 |

Only models with low differences in AICc values relative to the best model (Δi < 7) are shown. For each model, the predictor variable name (with its respective regression coefficient sign), Adjusted R², estimated number of parameters (K), AICc values, delta AICc and Akaike weights (AICw) are shown. The environmental variables were mean annual temperature (BIO1), temperature seasonality (BIO4), mean annual precipitation (BIO12), normalized index of vegetation difference (NDVI) and potential evapotranspiration (PET).


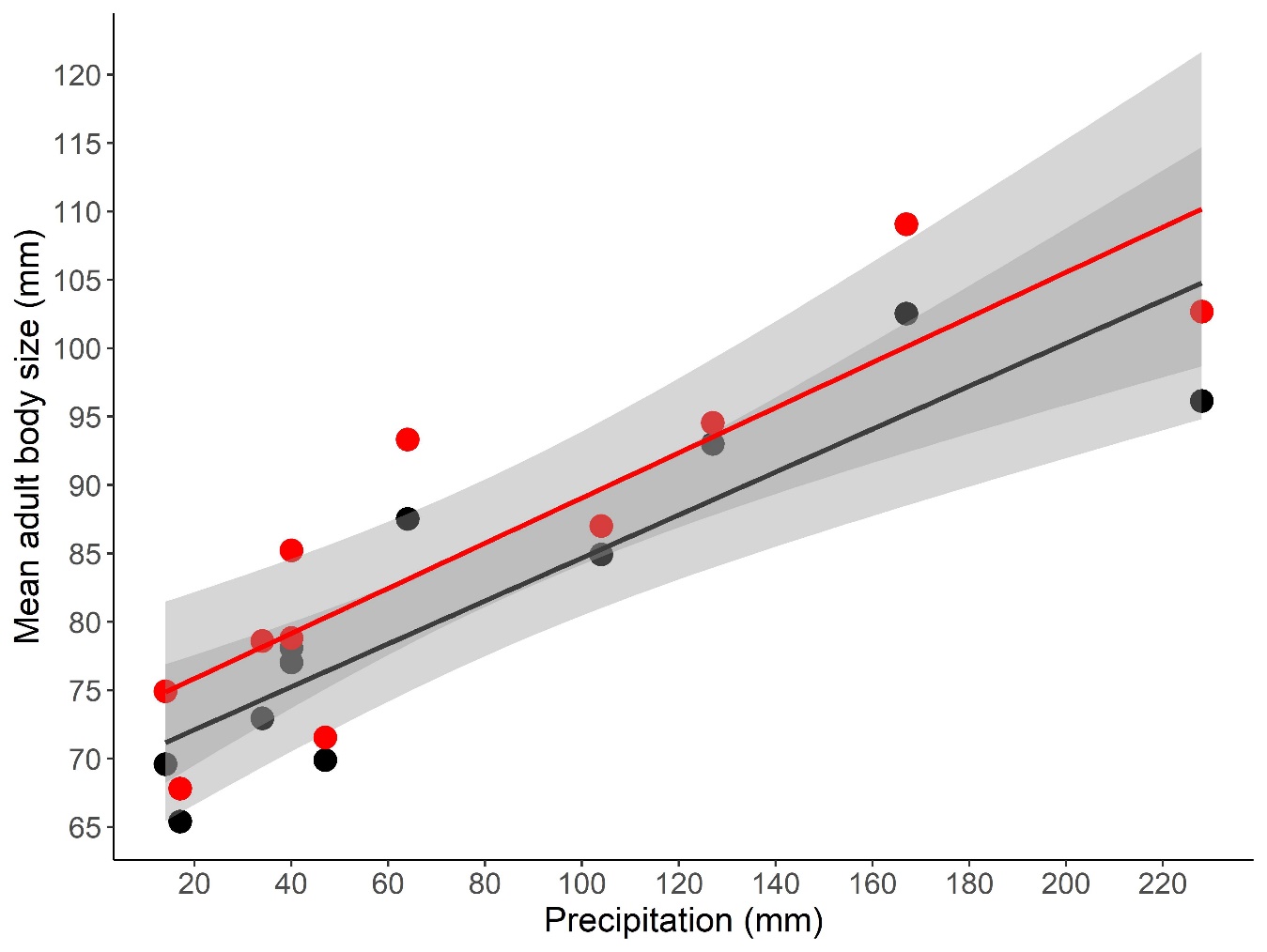


**Figure 1**. Relationship between mean adult body size (SVL) and precipitation of the 11 localities of Rhinella atacamensis studied. Red line and circles: females; black line and circles: males. The grey area indicates the 95% prediction interval of the mean. Localities are numbered according to Table 2.


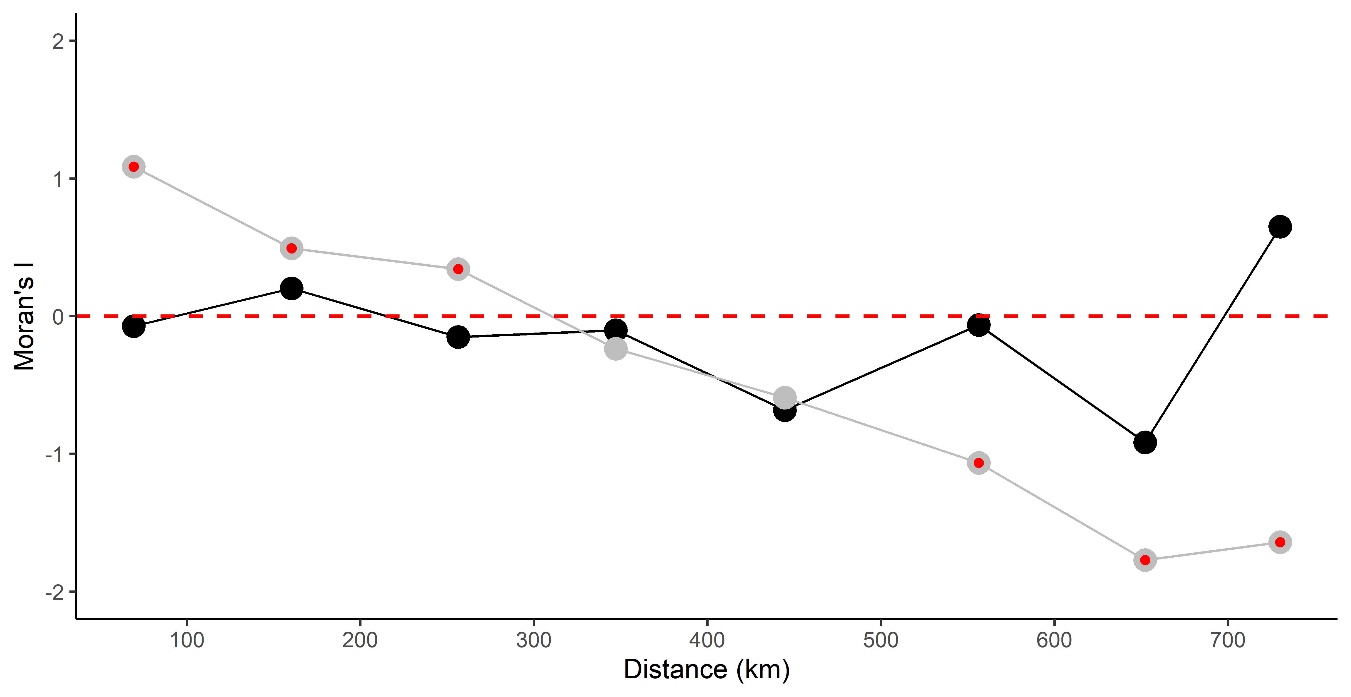


**Figure 2**. Spatial correlograms of Moran’s I of male adult mean SVL (grey circles and lines) and the residuals of the best model (black circles and lines) for eight distance classes. Red filled circles show statistical significance (p < 0.05).


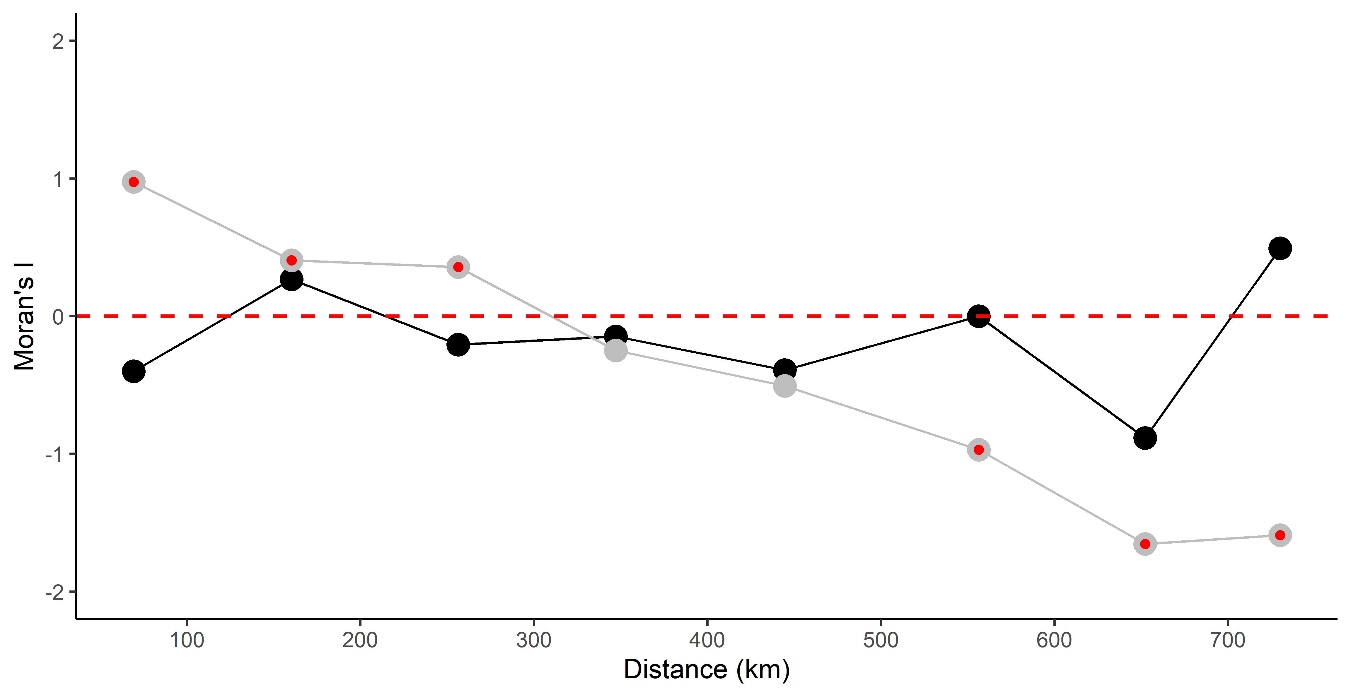


**Figure 3**. Spatial correlograms of Moran’s I of female adult mean SVL (grey circles and lines) and the residuals of the best model (black circles and lines) for eight distance classes. Red filled circles show statistical significance (p < 0.05).
